# Zinc and Breast Cancer Survival: A Prospective Cohort Study of Dietary Intake and Serum Levels

**DOI:** 10.3390/nu14132575

**Published:** 2022-06-22

**Authors:** Ylva Bengtsson, Kamil Demircan, Ann H. Rosendahl, Signe Borgquist, Malte Sandsveden, Jonas Manjer

**Affiliations:** 1 Department of Clinical Sciences Malmö, Lund University, 20213 Malmö, Sweden; malte.sandsveden@med.lu.se (M.S.); jonas.manjer@med.lu.se (J.M.); 2 Department of Surgery, Skåne University Hospital, 20501 Malmö, Sweden; 3 Institute for Experimental Endocrinology, Charité-Universitätsmedizin Berlin, Corporate Member of Freie Universität Berlin, Humboldt-Universität zu Berlin and Berlin Institute of Health, D-10115 Berlin, Germany; kamil.demircan@charite.de; 4 Berlin Institute of Health (BIH), Biomedical Innovation Academy (BIA), D-10117 Berlin, Germany; 5 Department of Clinical Sciences Lund, Oncology, Lund University, Skåne University Hospital, 22184 Lund, Sweden; ann.rosendahl@med.lu.se; 6 Department of Oncology, Aarhus University Hospital, Aarhus University, 8200 Aarhus, Denmark; signe.borgquist@auh.rm.dk

**Keywords:** zinc, breast cancer, survival, cohort, phosphorus, selenium

## Abstract

Zinc has been suggested to play a role in breast cancer progression; however, no previous study on zinc levels and the potential effect on breast cancer survival has been conducted. This study investigates recurrence-free survival (RFS), breast cancer-specific survival (BCSS) and overall survival (OS) in relation to zinc levels, in serum and diet, overall and stratified for phosphorus and selenium levels. The Malmö Diet and Cancer Study, a prospective population-based cohort in Sweden including 17,035 women, was used to identify breast cancer patients diagnosed in the period 1991–2013. Diet was assessed by a validated modified diet history method. A Cox regression analysis yielded hazard ratios (HRs) with 95% confidence intervals adjusted for potential confounders. Out of 1062 patients with invasive breast cancer, 268 recurrences, 205 breast cancer deaths and 228 deaths from other causes were recorded. No overall associations were seen between zinc and RFS, BCSS or OS. However, in women with a high phosphorus intake, a higher BCSS and OS were seen in zinc intake Q2 to Q4 versus Q1; the adjusted HR was 0.41 (0.23–0.73) and 0.64 (0.41–1.00), respectively. The results indicate that the combination of intermediate/high zinc intake and high phosphorus intake may lead to a better breast cancer survival.

## 1. Background

Zinc is an essential mineral incorporated into at least 300 enzymes, and is involved in numerous signaling pathways important for, e.g., cell proliferation and differentiation, cell cycle regulation, apoptosis and redox regulation [1]. While some reports exist on zinc levels and breast cancer risk [2,3,4], little is known about zinc regarding its potential effect on breast cancer survival. Although the potential role of zinc in breast cancer survival is not well-known, many possible biochemical mechanisms have been discussed [5]. Zinc has been reported in preclinical studies to trigger an interplay of G protein estrogen receptor with insulin-like growth factor receptor I (IGF-IR) and epidermal growth factor receptor, which results in the activation of important transduction pathways and biological responses such as proliferation and migration in breast cancer cells [6]. Furthermore, it has been shown that tamoxifen-resistant breast cancer cells have increased levels of zinc and zinc transporter ZIP7, leading to increased growth and invasion [7]. In addition, ZIP10 is involved in invasive behavior and metastasis of breast cancer cells [8].

One important aspect to consider when studying any essential nutrient is the possible interactions with other nutrients. Phosphorus, in the form of phytate, is common in vegetarian sources of zinc and has been shown to inhibit zinc absorption [1,9]. In addition, the balance between the trace element selenium and zinc has been suggested to play an important role in the onset of cancer [10]. Consequently, phosphorus and selenium levels may be important to take into consideration when studying zinc and breast cancer survival.

To our knowledge, no previous study on the potential effect of zinc levels on breast cancer survival has been conducted. However, several prospective epidemiological studies investigating the relationship between zinc and all-cause mortality reported either an inverse association [11,12,13,14] or no association at all [15,16]. Regarding cancer-specific mortality, Wu et al. (2004) found that cancer mortality was negatively related to serum zinc levels [14]. In contrast, Shi et al. (2017) found a positive association between relative zinc intake and cancer mortality [17].

The aim of this study was to investigate the potential effect of pre-diagnostic levels of zinc, in serum and diet, on breast cancer survival as measured with regard to recurrence-free survival (RFS), breast cancer-specific survival (BCSS) and overall survival (OS), overall and stratified for phosphorus and selenium levels, in a large prospective population-based cohort.

## 2. Materials and Methods

### 2.1. The Malmö Diet and Cancer Study

The study is based on women from the Malmö Diet and Cancer Study (MDCS), which was a prospective population-based cohort study conducted in Malmö, Sweden. Every citizen of Malmö born between 1923 and 1950 was invited to participate. A total of 28,098 individuals including 17,035 women were enrolled in the study, corresponding to a participation rate of 43% for women. The baseline examinations took place between 1991 and 1996 and included dietary assessment, blood samples and a self-administered questionnaire with questions regarding factors such as lifestyle, medical history, reproductive factors and socioeconomic status. The design and baseline examinations of the MDCS have been described in more detail elsewhere [18,19,20,21].

The present study was approved by the ethical committee in Lund, Sweden, (Dnr 2015/283). All participants signed an informed consent at baseline to allow collection of information, in addition to future follow-up (original ethical approval: LU 51-90).

### 2.2. Study Population

**Figure 1:**
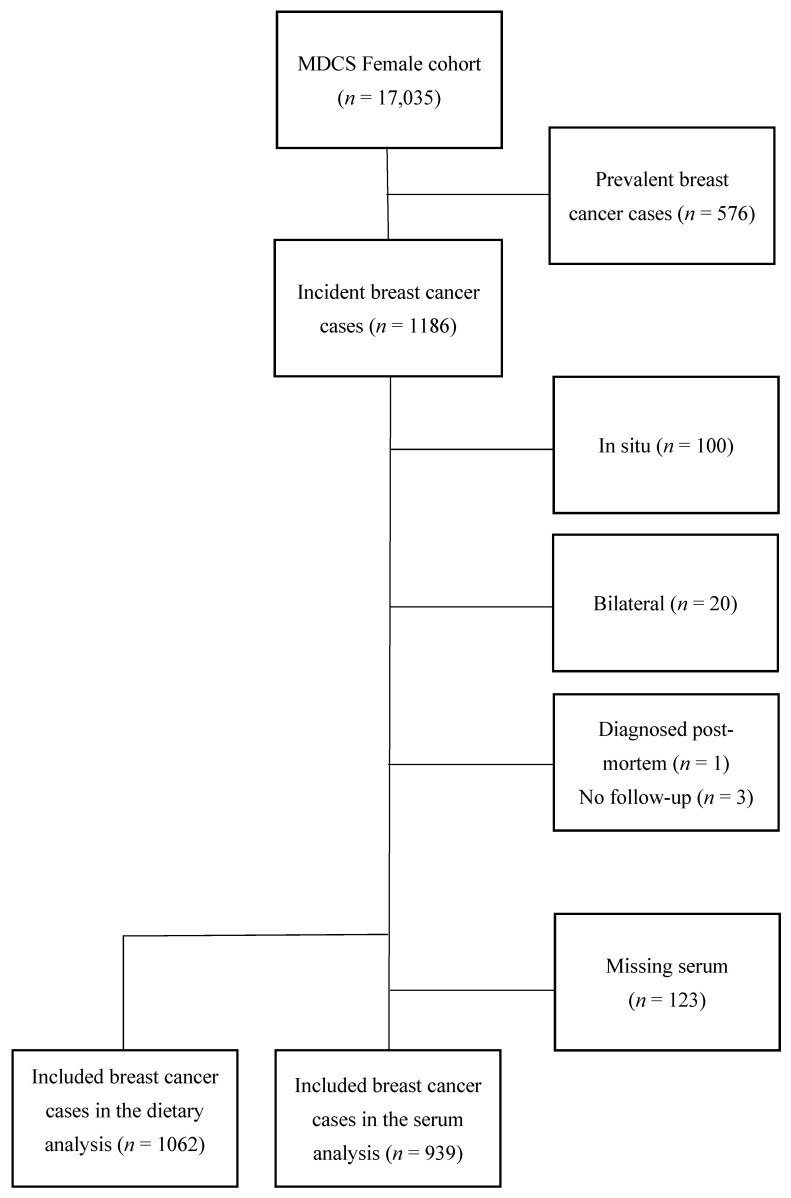
Flowchart of inclusion and exclusion.

Breast cancer cases were identified by record linkage with the Swedish Cancer Registry from 1991 up to 31 December 2013. Women who had been diagnosed with breast cancer prior to baseline examination (*n* = 576) were excluded. For the purpose of the current study, patients with carcinoma in situ were excluded (*n* = 100). In addition, incident cases with bilateral breast cancer (*n* = 20) were excluded due to difficulties in interpreting tumor characteristics. Finally, one woman who was diagnosed post mortem and three women who were not available for follow-up regarding recurrence status, but who were still alive according to the Swedish Cause of Death registry, were excluded. In the analyses based on serum zinc, women with an insufficient amount of saved serum (*n* = 123) could not be included. The final study population consisted of 939 women in the analyses using serum zinc and 1062 women in the analyses using dietary intake of zinc (Figure 1).

### 2.3. Dietary Data

The dietary assessment method used in the MDCS has been previously described [22]. In short, the method consists of (1) a 168-item diet history questionnaire for assessment of consumption frequencies, meal patterns and portion sizes of foods with low day-to-day variation, (2) a seven-day menu book for recording lunch and dinner meals, beverages, medications and nutrient supplements, and (3) a 45- to 60-min long interview where portion sizes and cooking practices in the menu book and questionnaire were specified in further detail. The food intake was translated into nutrient and energy intakes using PCKost2-93 from the National Food Administration in Uppsala, Sweden. The dietary intake of zinc was expressed as the sum of food intake and supplemental intake of zinc.

In September 1994, an unforeseen reduction of grants prompted a simplification of the interview routines in order to reduce interview time. However, the data are comparable, and the impact of the alteration has been shown to be small [22].

### 2.4. Laboratory Methods

Serum was extracted from non-fasting participants at baseline and stored at −80 °C until use. The analyses of serum zinc, selenium and phosphorus are described in more detail in three previous studies [4,21,23]. Briefly, analyses of zinc and selenium were conducted with an amount of 0.15 mL serum using inductively coupled plasma sector-field mass spectrometry. Phosphorus was analyzed using a colorimetric method by complexing with ammonium molybdate and creatinine. Inter-batch coefficients of variation were 3.3% for zinc, 3.4% for selenium, and 3.0% for phosphorus.

### 2.5. Endpoint Retrieval

All women were followed from the date of diagnosis until the end of follow-up and were censored if death or loss of follow-up (emigration) occurred. In the analysis using recurrent disease as the endpoint, patients were also censored if recurrence occurred. Overall and breast cancer-specific mortality data were collected from the Swedish Cause of Death registry by record-linking to the MDCS using the personal identity number. Breast cancer-specific mortality was defined as breast cancer being an underlying or contributing cause of death.

Data on recurrent disease were collected from medical records and pathology and radiology reports. This work was performed by registered nurses accustomed to monitoring clinical studies. When needed, ambiguous findings were discussed and finally classified in collaboration with a senior consultant in breast surgery (JM). Recurrent disease was defined as local, regional or distant recurrence, or death from breast cancer. Contralateral breast cancer was, regardless of whether it was defined as a new cancer or not, reported as distant metastasis. Three women had moved to another region in Sweden and, therefore, had no information regarding recurrent disease. All patients’ medical records were examined during spring 2020 to ascertain the date of the last clinical follow-up, and 31 December 2019 was subsequently used as the last date of follow-up for all patients.

### 2.6. Clinical Information and Histopathological Analysis

Collection of data on tumor characteristics was performed in three different time periods. In tumors from patients diagnosed until 31 December 2004, information on histological grade, estrogen receptor (ER)- and progesterone receptor (PgR) status, proliferation (Ki67) and human epidermal growth factor 2 (HER2) was re-evaluated on collected tumor samples, as previously described in more detail [24]. Similarly, for cases diagnosed from 2005 to 2007, a tissue micro array (TMA) was used to re-evaluate Ki67 and hormone receptor status [25]. From 2008 onwards, information about tumor characteristics was only collected from medical records. In addition, during all periods, medical records were used to gather information regarding tumor size and axillary lymph node involvement. HER2 data was collected from national registries, and if there were no conclusive data in the national registries, TMA or clinical records were used. No TMA data were used after 2005 for the HER2 variable [25]. Tumors were considered ER-positive (ER+) and PgR-positive (PgR+), according to the Swedish guidelines [26], if >10% of the nuclei were stained by immunohistochemistry. Based on the expression of Ki67, tumors were divided into tertiles (low, intermediate or high). Rankings were made separately for each period (1991–2004, 2005–2007 and 2008–2013) and afterwards merged into one variable.

Subsequently, as previously described [4], breast cancers were divided into four surrogate intrinsic subtypes: luminal A (ER+, HER2– and (1) grade 1 or (2) grade 2 and low Ki67 or (3) grade 2, intermediate Ki67 and PgR+); luminal B (ER+, HER2– and (1) grade 3 or (2) grade 2 and high Ki67 or (3) grade 2, intermediate Ki67 and PgR–); HER2-positive (all tumors regarded as HER2+ tumors); and triple negative breast cancer (TNBC) (ER–, PgR– and HER–).

### 2.7. Missing Values

The total amount of missing data among covariates included in the fully adjusted models using zinc intake and serum zinc made up 6.67% and 6.60% of all values, respectively. The variable with the most missing values was ‘intrinsic subtypes’ including 291 women (27.4%) with missing information in the analyses using zinc intake and 254 women (27.1%) in the analyses using serum zinc.

The missing values were imputed by chained equations. As recommended in a simulation study by Kruijk et al., an imputation model including the log of the survival time and an event indicator (in the current study, recurrent disease) was used [27]. Three different imputation models were created. In the first imputation model, we used zinc intake as an indicator of zinc status and imputed 25 new datasets, including 1062 women, using 10 iterations each. The following variables were included: zinc intake (quartiles), Log(t) (a logarithm with base 10 of time from diagnosis to recurrence/censoring), recurrence status (yes and no), age at baseline (continuous), age at diagnosis (continuous), baseline year (1991 to 1996), tumor size (≤10 mm, 11–20 mm, 21–50 mm, and >50 mm), lymph node status (yes and no), distant metastasis status (yes and no), intrinsic subtypes (luminal A, luminal B, HER2– and TNBC), surgical treatment (mastectomy, partial mastectomy, and local excision or surgical biopsy), hormone therapy (yes and no), radiotherapy (yes and no) and chemotherapy (yes and no). Due to missing values above 90%, information on neoadjuvant therapy was not imputed. In the second imputation model, we used serum zinc as an indicator of zinc status and imputed 25 new datasets, including only women with information on serum zinc levels (*n* = 939), using 10 iterations each. The imputation model included serum zinc (quartiles) and all of the abovementioned variables except zinc intake. The third imputation model was identical to the second imputation model, except that it only included women with information on serum phosphorus levels (*n* = 581). The pooled imputed data and the original data are shown in Supplementary Table S1. The robustness of the imputations was evaluated in a sensitivity analysis with only complete cases, which yielded similar results.

### 2.8. Statistical Analyses

Zinc intake was adjusted for energy intake by using the residual method, regressing total zinc intake on total energy intake. The study population was then divided into quartiles (Q) according to their energy-adjusted zinc intake and serum zinc levels. Quartiles of residuals are presented as the median and interquartile range (IQR) of total dietary intake of zinc.

Kaplan–Meier curves and logrank tests were used to visualize prognosis and to assess the proportional hazards assumption. Cox’s proportional hazard model was used to describe the association between quartiles of zinc, in diet and serum, and RFS, BCSS and OS. Multivariable adjustments were made for age at baseline, age at diagnosis, baseline year, lymph node status, tumor size, distant metastasis status at diagnosis and intrinsic subtype. Subsequently, linear trends among quartiles were tested by modeling the ordinal quartile variable as continuous. Following the analysis and discovering a potential threshold effect, all analyses were performed by merging Q2, Q3 and Q4 and comparing them to Q1.

The analyses merging zinc intake Q2 to Q4 versus Q1 were then stratified, using the median as cut-off, for energy-adjusted selenium intake (Q1 to Q2 and Q3 to Q4) and for energy-adjusted phosphorus intake (Q1 to Q2 and Q3 to Q4). Likewise, the analyses merging serum zinc Q2 to Q4 versus Q1 were stratified for serum selenium levels (Q1 to Q2 and Q3 to Q4), and a subsample, consisting of 581 cases, was stratified for serum phosphorus levels (Q1 to Q2 and Q3 to Q4). Moreover, by including the respective multiplicative term in the Cox regression model, interaction analyses were performed for: zinc intake and phosphorus intake, zinc intake and selenium intake, serum zinc and serum selenium, and, for the abovementioned subsample, serum zinc and serum phosphorus. The interaction analyses are presented with a *p*-value for interaction (P_i_).

Finally, several sensitivity analyses were performed. The first sensitivity analysis excluded patients diagnosed with breast cancer within the first year following baseline (*n* = 34). Secondly, additional analyses excluded women with an event/censoring within the first year of follow-up (*n* = 20 and *n* = 17). In a third analysis, additional adjustments were made for the interviewer who conducted the dietary interview, the season of collection of dietary data, and the dietary method before and after 1st September 1994, but not adjusting for baseline year. Furthermore, all analyses were repeated, excluding women reporting substantial diet changes prior to baseline (*n* = 278). Women with potentially unstable dietary habits were identified by the questionnaire item ‘Have you substantially changed your eating habits because of illness or some other reasons?’ In addition, all analyses were performed for zinc intake only from foods, excluding zinc intake from supplements. Moreover, a sensitivity analysis was conducted looking at 5-year and 10-year survival, respectively. Lastly, an additional analysis was performed, dichotomizing the follow-up time (using median as cut-off), studying women with a short time between baseline and diagnosis and women with a long time between baseline and diagnosis, separately.

All statistical analyses were conducted using SPSS Statistics version 25.

## 3. Results

Patient and tumor characteristics in relation to vital and relapse status are presented in Table 1. Out of 1062 patients with invasive breast cancer, 268 recurrences, 205 breast cancer deaths and 228 deaths from other causes were recorded. Women who died from breast cancer were older at baseline and diagnosis, and more frequently had larger tumors, positive lymph node status, distant metastasis at diagnosis, and luminal B-like, HER2+ and TNBC tumors compared to the women who were alive. Supplementary Table S2 presents treatment methods in relation to vital and recurrence status. Women who had died from breast cancer were more likely to have undergone mastectomy and chemotherapy. Women with recurrent disease had tumor and treatment characteristics similar to those of the women who died from breast cancer.

In Table 2 and Table S3, patient and tumor characteristics for different quartiles of zinc intake and serum zinc are presented. Women with the highest intake of zinc (Q4) were more likely to be classified as grade 1 and to take zinc supplements compared to those having the lowest zinc intake (Q1). However, women with the highest serum zinc levels (Q4) more frequently had smaller tumors and were more likely to be classified as grade 2 and to be PgR+ compared to women with the lowest serum zinc levels (Q1). Women with missing serum zinc levels were slightly older at baseline.

**Figure 2:**
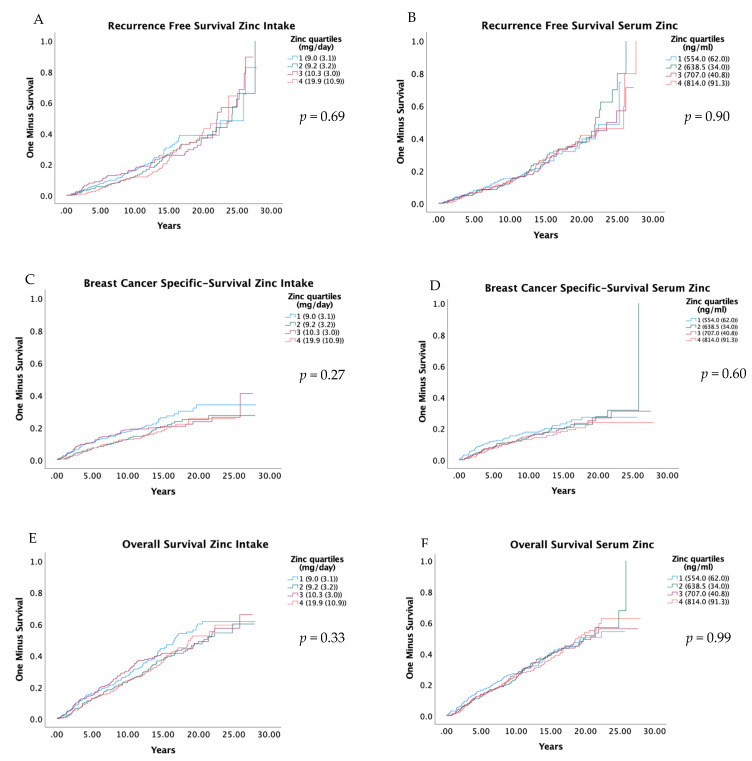
Kaplan-Meier curves for recurrence free survival (**A**,**B**), breast cancer-specific survival (**C**,**D**), and overall survival (**E**,**F**), by quartiles of zinc intake and serum zinc. Log-Rank-Test was used to evaluate differences. Residuals are presented as the median and interquartile range of total dietary intake of zinc. Quartiles of serum zinc are shown as median and interquartile range.

RFS, BCSS and OS were compared in relation to quartiles of zinc intake and serum zinc with Kaplan–Meier curves, and the results are presented in Figure 2. The results from univariate and adjusted Cox regression models are presented in Table 3. Zinc in the diet and serum were not associated with RFS or OS. However, a relatively low HR was seen in Q2, Q3 and Q4 compared to the first for BCSS, but these associations were not statistically significant; the adjusted HR for zinc intake Q2 versus Q1 was 0.75 (0.49–1.14) (P_trend_ 0.36), and for serum zinc Q2 versus Q1, it was 0.75 (0.50–1.15) (P_trend_ 0.33). Similarly, the abovementioned suggested pattern was also seen for the dichotomized groups; the adjusted HR for zinc intake Q2 to Q4 versus Q1 was 0.81 (0.58–1.13) (Table 4), and for serum zinc Q2 to Q4 versus Q1, it was 0.79 (0.56–1.12) (Table S4).

When the data were stratified for selenium levels, no associations between zinc (in diet or serum) and RFS, BCSS or OS were found (Table 4 and Table S4). Similarly, no significant interactions were found between zinc intake and different levels of selenium intake for RFS, BCSS and OS (Table 4 and Table S4).

When the data were stratified for different levels of phosphorus intake, no association between zinc intake and RFS was seen (Table 4). However, in women with a high phosphorus intake (above the median), a higher BCSS was seen in the dichotomized group of zinc intake Q2 to Q4 versus Q1; the adjusted HR was 0.41 (0.23–0.73) (P_i_ = 0.01) (Table 4). Likewise, the adjusted HR for OS in zinc intake Q2 to Q4 versus Q1 was 0.64 (0.41–1.00) (P_i_ = 0.10) (Table 4). When using serum zinc as an indicator of zinc status and stratifying for serum phosphorus levels, no associations with RFS, BCSS or OS were seen (Table S4).

When excluding cases during the first year following baseline, all HRs were similar to the main analyses (data not shown). Likewise, when excluding women with an event/censoring within the first year of follow-up, similar results were seen; the adjusted HR:s for BCSS in zinc intake Q4 versus Q1 was 0.76 (0.50–1.14), and in serum zinc Q4 versus Q1, it was 0.79 (0.50–1.26). In addition, further adjustments for dietary method, interviewer and season in the multivariate analyses using zinc intake as an indicator of zinc status did not alter the results notably; the adjusted HR for BCSS in zinc intake Q4 versus Q1 was 0.72 (0.45–1.11). When excluding women who had reported substantial change in dietary habits, similar results were seen; the adjusted HR for BCSS in zinc intake Q4 versus Q1 was 0.78 (0.49–1.25) Furthermore, when the analyses were made for zinc intake only from foods, the results were slightly altered, with the adjusted HR for BCSS in Q1: 1.00 (reference), Q2: 0.69 (0.46–1.05), Q3: 0.75 (0.50–1.12) and Q4: 0.78 (0.52–1.15) (Table S5). Zinc in relation to RFS, BCSS and OS, stratified for time between baseline and diagnoses, is presented in Supplementary Table S6, and zinc in relation to 5-year and 10-year RFS, BCSS and OS is presented in Supplementary Table S7.

## 4. Discussion

To the best of our knowledge, this is the first study to assess the potential effect of pre-diagnostic zinc on breast cancer survival. No overall associations were seen between zinc and RFS, BCSS or OS. However, better BCSS and OS were seen for intermediate/high zinc intake in the group with high phosphorus intake.

Previous studies investigating the relationship between zinc and all-cause- or cancer-specific mortality have rendered mixed results. A study of a national cohort from the United States, including 6244 individuals, found that serum zinc was negatively related to cancer mortality [14]. In addition, the Paris Prospective Study 2, including more than 4000 men, suggests that a combination of low serum zinc and high serum copper or low magnesium results in an increased cancer- and all-cause mortality risk [11]. In contrast, a study in Finland among 344 elderlies found no association between serum zinc and all-cause mortality; however, these results might be limited by the relatively low number of participants [15]. Moreover, a study in Jiangsu Province, China, including 2832 adults, found a positive association between zinc intake and all-cause and cancer mortality [17]. Consequently, similarly to our results, previous research suggests that there might be a potential association between zinc and breast cancer prognosis, even though the evidence remains inconclusive.

Our study showed better BCSS and OS for intermediate/high zinc intake in the group with high phosphorus intake. It is well–known that phosphorus, in the form of phytate, inhibits zinc absorption by forming insoluble complexes in the gastrointestinal tract that cannot be absorbed due to the absence of intestinal phytase enzymes [1,9]. Indeed, a meta-analysis by Bel-Serrat et al. (2014), including 30 studies, revealed an overall reduction of fractional zinc absorption by 45% of the control meals when the phytate/zinc molar ratio of the diet was greater than 15 [28]. In addition to phosphorus, other factors have been identified to have a possible effect on serum/plasma zinc levels, such as time of day [29], albumin levels [30] and infection [31]. It can be hypothesized that an effect of zinc on breast cancer prognosis might be seen only when zinc levels are reduced by external factors. Since our study was the first to take phosphorus intake into account when evaluating the association between zinc and breast cancer survival, future studies should consider the possible interaction between zinc and phosphorus, as well as other factors affecting zinc levels.

Our study has several strengths. MDCS is a large and well-characterized population-based prospective observational study with a relatively long follow-up. Moreover, data on tumor characteristics were collected, which enabled adjustment for many potential confounders, even though residual confounding cannot be ruled out.

Concerning the risk of a potential selection bias, the participation rate for women in the MDCS was 43%, but previous analyses have shown that the MDCS had sociodemographic characteristics and prevalence of obesity and smoking similar to those of the overall background population [20]. In addition, the mean total daily zinc intake in our study (12.1 ± 0.2 mg/day) was close to the mean total daily zinc intake for women in the National Health and Nutrition Examination Survey in the US 2011–2014 (13.4 ± 0.4 mg/day) [30].

Another strength is the use of two different indicators of zinc status. The modified diet history methodology used in the MDCS was especially developed to reflect the usual intake of individuals, and the relative validity and reproducibility of this methodology has proved to be high [32,33]. In the validation study, a slightly different dietary assessment method (a 2-week food record and a 130-item questionnaire) was compared against a reference method of 18-day weighted food records collected over 1 year. The energy-adjusted correlation coefficients for zinc and selenium were 0.44 and 0.44, respectively [32]. Furthermore, a sensitivity analysis excluding women reporting substantial diet changes prior to baseline did not alter the results notably. In addition, the inter-batch coefficients of variation for the serum analyses were 3.3% for zinc, 3.0% for phosphorus and 3.4% for selenium, which increased the reliability of our measurements. Taken together, these points show there is a low risk of misclassification bias regarding the exposure variable, zinc status.

Besides using two different indicators of zinc status, the Swedish Cause of Death registry is a high-quality, virtually complete register on the event of death, and 96% of individuals in the registry have a specific underlying cause of death recorded [34]. Furthermore, the registry has been shown to be correct in approximately 90% of cases where malignant neoplasms were the cause of death [35]. Consequently, data regarding cause of death in Sweden are expected to be both complete and correct to a large extent.

One limitation of the study is that serum sampling was only performed once, from a single blood sample taken pre-diagnostically. Thus, circumstantial factors, such as a zinc-enriched meal, time of day, albumin levels and infection, might affect the acute zinc status. However, it has been suggested that strong homeostatic mechanisms exist to prevent deviations in serum zinc when dietary intakes fluctuate, which might help in maintaining long-term ranking between individuals [30,36].

Although serum/plasma zinc concentration and dietary zinc intake are recommended as biomarkers of zinc status by Biomarkers of Nutrition for Development (BOND) Zinc Expert Panel, the search for a more reliable indicator for zinc continuous [1]. Several potential emerging biomarkers of zinc status have been identified, e.g., concentrations of zinc metalloenzymes and zinc-binding proteins, plasma zinc turnover rates and zinc concentrations in nail, hair and urine. However, further research is needed before those biomarkers can be used to determine the zinc status of individuals or a population. Moreover, our results from a previous study of the MDCS showed a poor agreement between serum zinc and zinc intake with a kappa value of 0.03 (*p* = 0.02) [4]. This is in line with the National Health and Nutrition Examination Survey 2011–2014, including 4347 individuals in the US, showing that serum zinc levels were not related to zinc intake [30].

Further limitations include the risk of type I errors due to multiple comparisons. However, the analyses with zinc intake pointed in the same direction as the analyses with serum zinc, which strengthens the evidence that the findings could be due to a true effect rather than chance. In addition, we did find significant results in the interaction analyses indicating that the power was high enough to detect a difference. The risk of a type II error must also be considered, as the statistical power in some of the stratified analyses, and some sensitivity analyses, was limited. This is also a problem considering that we included a long time period, and at the end of the period, survival curves will be less reliable due to the low number of patients and events.

## 5. Conclusions

This study found no overall evidence of an effect of pre-diagnostic zinc on recurrence-free, breast cancer-specific or overall survival. However, better breast cancer-specific and overall survival were seen for intermediate/high zinc intake in the group with high phosphorus intake.

## Figures and Tables

**Table 1:** Vital status and prognostic factors. All data are presented as column percentage, except for mean serum zinc, zinc intake and age. Missing data <1% are not shown.

		Alive (*n* = 629)	Recurrent Disease (*n* = 268)	Breast Cancer Death (*n* = 205)	Other Death (*n* = 228)	Total (*n* = 1062)
Mean (SD ^a^) serum zinc (ng/mL)		677.4 (126.3)	686.8 (120.5)	677.4 (123.3)	687.5 (117.3)	679.5 (123.9)
Mean (SD ^a^) dietary zinc intake (mg/day)		12.3 (5.8)	11.7 (5.4)	11.6 (5.5)	12.2 (6.0)	12.1 (5.8)
Mean (SD ^a^) age at baseline		53.7 (5.9)	56.3 (6.8)	58.7 (7.8)	61.4 (6.9)	56.3 (7.3)
Mean (SD ^a^) age at diagnosis		64.9 (7.7)	64.9 (8.5)	67.8 (9.9)	70.7 (8.4)	66.7 (8.6)
Baseline year	1991	8.1	8.6	9.8	10.4	9.0
	1992	20.5	19.4	21.0	18.5	19.7
	1993	19.6	19.8	19.5	18.9	19.3
	1994	18.6	18.7	14.1	17.8	18.3
	1995	20.5	22.8	22.4	21.7	21.0
	1996	12.7	10.8	13.2	12.7	12.7
Tumor size	≤10.00 mm	29.6	16.4	5.9	24.1	23.8
	10.01–20.00 mm	47.4	42.5	33.7	44.7	44.2
	20.01–50.00 mm	19.7	32.1	40.5	23.7	24.6
	>50.01 mm	1.1	6.3	9.3	3.9	3.3
	Missing	2.2	2.6	10.7	3.5	4.1
Lymph node status	Positive	22.1	43.3	53.2	24.6	28.6
	Negative	70.1	50.0	35.1	62.3	61.7
	Missing	7.8	6.7	11.7	13.2	9.7
Distant metastasis	Yes	0.0	0.7	6.8	0.0	1.3
	No	96.3	95.9	84.4	92.5	93.2
	Missing	3.7	3.4	8.8	7.5	5.5
Intrinsic subtypes	Luminal A	46.9	30.2	19.0	41.2	40.3
	Luminal B	16.4	23.1	24.9	18.4	18.5
	HER2+	6.4	7.8	10.2	7.9	7.4
	Triple negative	5.7	8.2	8.8	6.1	6.4
	Missing	24.6	30.6	37.1	26.3	27.4
ER	Positive	8.7	12.3	12.2	8.8	9.4
	Negative	81.4	71.3	63.4	80.7	77.8
	Missing	9.9	16.4	24.4	10.5	12.8
PgR	Positive	31.2	39.9	37.6	34.6	33.1
	Negative	56.1	39.6	35.1	50.4	50.8
	Missing	12.7	20.5	27.3	14.9	16.0
Histological grade	Grade 1	29.7	17.9	9.3	24.6	24.7
	Grade 2	45.5	37.7	35.1	46.5	43.7
	Grade 3	18.6	35.8	41.5	23.2	24.0
	Missing	6.2	8.6	14.1	5.7	7.6
Ki67	Low	33.4	24.6	19.0	25.0	28.8
	Intermediate	21.8	24.6	20.0	29.4	23.1
	High	16.9	28.0	32.2	24.1	21.4
	Missing	28.0	22.8	28.8	21.5	26.7

^a^ Standard deviation.

**Table 2:** Prognostic factors for breast cancer and zinc intake.

		Dietary Intake of Zinc ^a^				Total
		1 (*n* = 266)	2 (*n* = 265)	3 (*n* = 265)	4 (*n* = 266)	
		9.0 (3.1) mg/day	9.2 (3.2) mg/day	10.3 (3.0) mg/day	19.9 (10.9) mg/day	(*n* = 1062)
Mean (SD) age at baseline		56.7 (7.7)	56.3 (7.5)	55.7 (7.2)	56.6 (6.9)	56.3 (7.3)
Mean (SD) age at diagnosis		67.3 (8.5)	66.4 (9.2)	66.0 (8.4)	67.3 (8.2)	66.7 (8.6)
Zinc supplements	Yes	98.1	96.6	92.8	33.8	80.3
	No	1.9	3.4	7.4	66.2	19.7
Baseline year	1991	9.8	7.9	9.1	9.4	9.0
	1992	15.4	21.5	18.1	23.7	19.7
	1993	17.7	18.5	23.0	18.0	19.3
	1994	20.7	17.4	17.0	18.0	18.3
	1995	25.2	20.8	19.2	18.8	21.0
	1996	11.3	14.0	13.6	12.0	12.7
Tumor size	≤10.00 mm	23.7	20.0	24.9	26.7	23.8
	10.01–20.00 mm	40.2	50.6	42.6	43.2	44.2
	20.01–50.00 mm	22.9	25.7	24.9	24.8	24.6
	>50.01 mm	6.4	1.9	3.0	1.9	3.3
	Missing	6.8	1.9	4.5	3.4	4.1
Lymph node status	Positive	32.0	27.5	27.2	27.8	28.6
	Negative	56.4	64.9	64.2	61.3	61.7
	Missing	11.7	7.5	8.7	10.9	9.7
Distant metastasis	Yes	1.9	1.1	0.8	1.5	1.3
	No	92.1	94.0	92.5	94.4	93.2
	Missing	6.0	4.9	6.8	4.1	5.5
Intrinsic subtypes	Luminal A	42.9	36.6	38.5	43.2	40.3
	Luminal B	18.4	18.1	18.1	19.2	18.5
	HER2+	4.9	10.9	9.1	4.9	7.4
	Triple negative	3.4	10.2	7.2	4.9	6.4
	Missing	30.5	24.2	27.2	27.8	27.4
ER	≤10	6.0	12.8	10.6	8.3	9.4
	>10	78.6	74.3	78.9	79.3	77.8
	Missing	15.4	12.8	10.6	12.4	12.8
PgR	≤10	32.0	34.7	34.3	31.6	331
	>10	50.0	48.7	50.6	54.1	50.8
	Missing	18.0	16.6	15.1	14.3	16.0
Histological grade	Grade 1	23.3	24.5	21.5	29.3	24.7
	Grade 2	43.2	43.8	45.7	42.1	43.7
	Grade 3	22.9	27.5	25.3	20.3	24.0
	Missing	10.5	4.2	7.5	8.3	7.6
Ki67	Low	27.1	27.2	32.1	28.9	28.8
	Intermediate	25.9	21.9	17.7	26.7	23.1
	High	16.5	26.0	22.6	20.3	21.4
	Missing	30.5	24.9	27.5	24.1	26.7

All data are presented as column percentage, except for age, which is presented as mean years and standard deviation (SD). Missing data ≤1% are not shown. ^a^ Residuals are presented as the median and interquartile range of total dietary intake of zinc.

**Table 3:** Zinc in diet and serum in relation to recurrence-free survival (RFS), breast cancer-specific survival (BCSS) and overall survival (OS).

		Dietary Intake of Zinc ^a^						Serum Zinc ^a^					
	Zinc Quartile	Women (*n*)	Events (*n*)	Total Person Years	Mortality/1000	HR (95% CI)	HR (95% CI) ^b^	Women (*n*)	Events (*n*)	Total Person Years	Mortality/1000	HR (95% CI)	HR (95% CI) ^b^
RFS	1	266	67	2983	22.46	1.00	1.00	236	52	2616	19.88	1.00	1.00
	2	265	68	3296	20.63	0.87 (0.62–1.22)	0.91 (0.64–1.29)	238	60	2761	21.73	1.08 (0.75–1.57)	0.95 (0.65–1.39)
	3	265	73	3133	23.30	1.00 (0.76–1.39)	1.06 (0.75–1.50)	232	57	2827	20.16	0.95 (0.65–1.39)	0.93 (0.63–1.37)
	4	266	60	3219	18.64	0.85 (0.60–1.20)	0.90 (0.63–1.28)	233	61	2860	21.33	0.99 (0.68–1.44)	1.01 (0.69–1.49)
	P-trend					0.55	0.79					0.77	0.97
BCSS	1	266	59	3040	19.41	1.00	1.00	236	48	2692	17.83	1.00	1.00
	2	265	48	3365	14.26	0.74 (0.51–1.09)	0.75 (0.49–1.14)	238	45	2817	15.97	0.90 (0.60–1.35)	0.75 (0.50–1.15)
	3	265	53	3216	16.48	0.86 (0.59–1.24)	0.94 (0.63–1.42)	232	44	2875	15.30	0.86 (0.57–1.30)	0.84 (0.55–1.28)
	4	266	45	3210	14.02	0.70 (0.45–1.04)	0.76 (0.50–1.14)	233	39	2938	13.27	0.75 (0.49–1.14)	0.77 (0.49–1.20)
	P-trend					0.14	0.36					0.20	0.33
OS	1	266	114	3040	37.50	1.00	1.00	236	89	2692	33.06	1.00	1.00
	2	265	103	3365	30.61	0.80 (0.62–1.05)	0.85 (0.64–1.13)	238	92	2817	32.66	0.99 (0.74–1.32)	0.84 (0.62–1.12)
	3	265	113	3216	35.14	0.92 (0.71–1.20)	1.03 (0.78–1.36)	232	92	2875	32.00	0.96 (0.72–1.28)	0.91 (0.68–1.23)
	4	266	103	3210	32.09	0.82 (0.63–1.08)	0.90 (0.68–1.19)	233	96	2938	32.68	0.98 (0.72–1.30)	0.92 (0.68–1.24)
	P-trend					0.31	0.78					0.82	0.76

^a^ Serum zinc quartiles and quartiles of dietary intake of zinc as in Table 2. ^b^ Adjusted for age at baseline, age at diagnosis, baseline year, tumor size, lymph node status, distant metastasis status and intrinsic subtype.

**Table 4:** Zinc intake in relation to recurrence-free survival (RFS), breast cancer-specific survival (BCSS) and overall survival (OS).

				Dietary Intake of Zinc ^a^					
			Zinc Quartile	Women (*n*)	Events (*n*)	Total Person Years	Mortality/1000	HR (95% CI)	HR (95% CI) ^b^
All		RFS	1	266	67	2983	22.46	1.00	1.00
			2 + 3 + 4	796	201	9648	20.83	0.91 (0.69–1.20)	0.96 (0.72–1.27)
		BCSS	1	266	59	3040	19.41	1.00	1.00
			2 + 3 + 4	796	146	9791	14.91	0.77 (0.57–1.04)	0.81 (0.58–1.13)
		OS	1	266	114	3040	37.50	1.00	1.00
			2 + 3 + 4	796	319	9791	32.58	0.85 (0.69–1.05)	0.92 (0.74–1.16)
Phosphorus intake ^c^	Low	RFS	1	127	30	1415	21.20	1.00	1.00
			2 + 3 + 4	404	107	4721	22.66	0.98 (0.69–1.38)	1.02 (0.72–1.46)
		BCSS	1	127	28	1441	19.43	1.00	1.00
			2 + 3 + 4	404	71	4831	14.70	0.88 (0.59–1.32)	1.04 (0.67–1.62)
		OS	1	127	58	1441	40.25	1.00	1.00
			2 + 3 + 4	404	156	4831	32.29	0.92 (0.70–1.20)	1.02 (0.76–1.37)
	High	RFS	1	139	40	1616	24.75	1.00	1.00
			2 + 3 + 4	392	91	4880	18.44	0.89 (0.48–1.65)	0.87 (0.46–1.66)
				P_i_^d^ high versus low phosphorus				0.87	0.81
		BCSS	1	139	31	1655	18.73	1.00	1.00
			2 + 3 + 4	392	75	4994	15.02	0.48 (0.28–0.82)	0.41 (0.23–0.73)
				P_i_ high versus low phosphorus				0.07	0.01
		OS	1	139	68	1655	41.09	1.00	1.00
			2 + 3 + 4	392	151	4994	30.24	0.69 (0.45–1.06)	0.64 (0.41–1.00)
				P_i_ high versus low phosphorus				0.28	0.10
Selenium intake ^c^	Low	RFS	1	91	29	1010	28.71	1.00	1.00
			2 + 3 + 4	440	113	5288	21.37	0.66 (0.44–1.00)	0.77 (0.49–1.19)
		BCSS	1	91	21	1023	20.53	1.00	1.00
			2 + 3 + 4	440	86	5419	15.87	0.78 (0.48–1.25)	0.99 (0.58–1.68)
		OS	1	91	41	1023	40.08	1.00	1.00
			2 + 3 + 4	440	160	5419	29.53	0.72 (0.51–1.02)	0.91 (0.63–1.33)
	High	RFS	1	175	41	2020	20.30	1.00	1.00
			2 + 3 + 4	356	85	4313	19.71	0.93 (0.64–1.36)	1.09 (0.73–1.62)
				P_i_ high versus low selenium				0.25	0.22
		BCSS	1	175	38	2073	18.33	1.00	1.00
			2 + 3 + 4	356	60	4405	13.62	0.75 (0.50–1.13)	0.99 (0.64–1.55)
				P_i_ high versus low selenium				0.87	0.80
		OS	1	175	85	2073	41.00	1.00	1.00
			2 + 3 + 4	356	147	4405	33.37	0.82 (0.62–1.07)	1.07 (0.80–1.43)
				P_i_ high versus low selenium				0.59	0.40

^a^ Quartiles of dietary intake of zinc as in Table 2. ^b^ Adjusted for age at baseline, age at diagnosis, baseline year, tumor size, lymph node status, distant metastasis status and intrinsic subtype. ^c^ The cut-off is set at the median. Residuals are presented as the median and interquartile range of total dietary intake of phosphorus and selenium, with low defined as 1199 (388) ng/day and 28.06 (10.36) µg/day, respectively, and high defined as 1576 (467) ng/day and 50.10 (35.39) µg/day, respectively. ^d^ *p*-value for interaction.

## Data Availability

The data will be shared on reasonable request to the corresponding author.

## Supplementary Materials

The following supporting information can be downloaded at: https://www.mdpi.com/article/10.3390/nu14132575/s1, Table S1: Pooled imputed values and original values; Table S2: Mortality status and treatment amongst cases in main survival analysis; Table S3: Prognostic factors for breast cancer and serum zinc levels; Table S4: Serum zinc in relation to recurrence-free survival (RFS), breast cancer-specific survival (BCSS) and overall survival (OS); Table S5: Zinc intake from foods in relation to recurrence-free survival (RFS), breast cancer-specific survival (BCSS) and overall survival (OS). Table S6: Zinc in diet and serum in relation to recurrence-free survival (RFS), breast cancer-specific survival (BCSS) and overall survival (OS), stratified for time between baseline and diagnosis. Table S7: Zinc in diet and serum in relation to 5 and 10-year recurrence-free survival (RFS), breast cancer-specific survival (BCSS) and overall survival (OS).
