# Supplementary material for: Zinc and Breast Cancer Survival: A Prospective Cohort Study of Dietary Intake and Serum Levels
Source: Nutrients. 2022 Jun 22;14(13):2575. doi: 10.3390/nu14132575 (PMC9268140; doi:10.3390/nu14132575)
Supplement: Supplementary file 1 [file nutrients-14-02575-s001.zip › nutrients-1720293-supplementary.pdf]

nutrients

Supplementary Materials

Zinc and Breast Cancer Survival: A Prospective Cohort Study of Dietary Intake and Serum Levels

Ylva Bengtsson [1,2,*], Kamil Demircan [3,4], Ann H. Rosendahl [5], Signe Borgquist [6], Malte Sandsveden [1,2] and Jonas Manjer [1,2]

Table S1. Pooled imputed values and original values.

	Original (*n* = 1062)	Imputed[a] (*n* = 1062)	Imputed[b] (*n* = 939)	Imputed[c] (*n* = 582)
Baseline year				
1991	9.0	9.0	8.6	
1992	19.7	19.7	20.9	22.0
1993	19.3	19.3	18.4	20.0
1994	18.3	18.3	18.3	18.4
1995	21.0	21.0	20.9	19.8
1996	12.7	12.7	12.9	10.3
Tumor size				
≤10.00 mm	23.8	24.5	24.3	26.3
10.01-20.00 mm	44.2	45.5	46.4	45.5
20.01-50.00 mm	24.6	25.9	25.9	25.2
>50.01 mm	3.3	4.1	3.3	3.0
Missing	4.1			
Lymph node status				
Positive	28.6	30.9	31.5	32.2
Negative	61.7	69.1	68.5	67.8
Missing	9.7			
Distant metastasis				
Yes	1.3	3.9	1.5	1.6
No	93.2	96.1	98.5	98.4
Missing	5.5			
Intrinsic subtype				
Luminal A	40.3	54.8	54.6	56.2
Luminal B	18.5	25.6	25.5	23.9
HER2+	7.4	10.5	10.7	10.3
Triple negative	6.4	9.2	9.2	9.6
Missing	27.4			
Surgical treatment				
Mastectomy	34.6	42.8	41.0	40.9
Partial Mastectomy	61.1	55.2	55.0	57.9
Local excision or surgical biopsy	0.4	2.0	4.0	1.3
Missing	3.9			
Hormone therapy				
Yes	56.0	57.2	58.4	48.6
No	41.5	42.8	41.6	51.4
Missing	2.5			
Radiotherapy				
Yes	58.1	58.2	58.5	59.1
No	33.1	41.8	41.5	40.9
Missing	8.8			
Chemotherapy				
Yes	13.3	15.6	16.3	15.7
No	77.6	84.4	83.7	84.3
Missing	9.1			

All data are presented as column percentage. Missing not presented if no missing values.

[a] Multiple imputation performed using dietary intake of zinc as an indicator of zinc status.

[b] Multiple imputation performed using serum zinc as an indicator of zinc status.

[c] Multipe imputation performed using serum zinc as an indicator if zinc status for women with information on serum phosphorus levels.

Table S2. Mortality status and treatment amongst cases in main survival analysis.

	Alive (n = 629)	Recurrent disease (n = 268)	Breast cancer death (n = 205)	Other death (n = 228)	Total (n = 1062)
Surgical treatment					
Mastectomy	33.4	51.5	59.0	48.0	39.4
Partial Mastectomy	62.3	44.8	27.3	40.6	53.5
Local excision or surgical biopsy	0.5	0.4	1.5	0.9	0.7
Missing	3.8	3.4	12.2	10.4	6.5
Hormone therapy					
Yes	55.5	51.1	58.0	55.9	55.6
No	41.8	44.8	36.6	40.9	41.4
Missing	2.7	4.1	5.4	3.2	2.9
Radiotherapy					
Yes	58.3	58.6	52.7	43.9	54.9
No	32.4	32.1	40.5	49.9	37.1
Missing	9.2	9.3	6.8	6.2	8.0
Chemotherapy					
Yes	13.7	22.0	28.3	16.2	14.7
No	76.9	69.0	64.9	77.4	77.1
Missing	9.4	9.0	6.8	6.5	8.2
Neoadjuvant chemotherapy					
Yes	1.0	0.7	0.5	0.5	0.8
No	16.5	6.7	8.8	9.9	13.8
Missing	82.5	92.5	90.7	89.6	85.4

All data is presented as column percentage. Missing data is not presented if missing ≤1%. Data for neoadjuvant chemotherapy only available from 2008.

Table S3. Prognostic factors for breast cancer and serum zinc levels.

	Serum zinc[a]				Missing	Total
	**1** (*n* = 236)	**2** (*n* = 238)	**3** (*n* = 232)	**4** (*n* = 233)		
	**554.0 (62.0) ng/ml**	**638.5 (34.0) ng/ml**	**707.0 (40.8) ng/ml**	**814.0 (91.3) ng/ml**	**(*n* = 123)**	**(*n* = 939)**
Mean (SD) age at baseline	55.0 (7.4)	56.1 (7.8)	56.0 (7.4)	57.0 (6.5)	58.7 (6.9)	56.3 (7.3)
Mean (SD) age at diagnosis	66.4 (9.2)	66.9 (9.4)	66.3 (8.4)	67.3 (7.8)	66.7 (7.6)	66.7 (8.6)
Baseline year						
1991	7.2	6.3	9.1	12.0	12.2	9.0
1992	21.6	18.5	17.2	26.2	10.6	19.7
1993	15.3	14.3	23.3	21.0	26.0	19.3
1994	14.4	20.6	19.0	19.3	17.9	18.3
1995	23.3	26.5	18.5	15.0	22.0	21.0
1996	18.2	13.9	12.9	6.4	11.4	12.7
Tumor size						
≤10.00 mm	22.0	25.6	18.5	27.0	27.6	23.8
10.01-20.00 mm	45.3	40.3	47.0	45.5	41.5	44.2
20.01-50.00 mm	22.5	25.6	28.0	23.2	22.8	24.6
>50.01 mm	3.4	4.2	3.0	2.1	4.1	3.3
Missing	6.8	4.2	3.4	2.1	4.1	4.1
Lymph node status						
Positive	27.5	30.3	29.7	27.0	28.5	28.6
Negative	61.4	60.9	61.6	65.7	56.1	61.7
Missing	11.0	8.8	8.6	7.3	15.4	9.7
Distant metastasis						
Yes	2.5	0.8	0.4	1.7	0.8	1.3
No	91.5	91.2	93.5	94.0	98.4	93.2
Missing	5.9	8.0	6.0	4.3	0.8	5.5
Intrinsic subtypes						
Luminal A	39.0	40.8	43.1	36.5	43.9	40.3
Luminal B	17.4	18.5	19.8	18.9	17.1	18.5
HER2+	9.3	8.8	5.2	7.7	4.9	7.4
Triple negative	7.2	5.9	3.0	10.7	4.1	6.4
Missing	27.1	26.1	28.9	26.2	30.1	27.4
ER						
Positive	8.9	8.8	6.9	13.3	8.9	9.4
Negative	76.3	79.8	80.2	76.0	75.6	77.8
Missing	14.8	11.3	12.9	10.7	15.4	12.8
PgR						
Positive	36.0	31.1	27.6	33.9	40.7	33.1
Negative	45.3	54.6	56.0	51.5	43.1	50.8
Missing	18.6	14.3	16.4	14.6	16.3	16.0
Histological grade						
Grade 1	26.3	23.9	27.2	18.9	29.3	24.7
Grade 2	42.4	41.6	42.2	49.8	41.5	43.7
Grade 3	22.0	25.6	23.3	25.8	22.8	24.0
Missing	9.3	8.8	7.3	5.6	6.5	7.6
Ki67						
Low	30.5	29.4	25.0	28.8	31.7	28.8
Intermediate	22.0	21.4	24.6	22.7	26.0	23.1
High	19.9	20.2	20.3	24.9	22.0	21.4
Missing	27.5	29.0	30.2	23.6	20.3	26.7

All data are presented as column percentage, except for age which is presented as mean years and standard deviation (SD). Missing data <1% is not shown.

[a] Quartiles of serum zinc are shown as median and interquartile range.

Table S4. Serum zinc in relation to recurrence free survival (RFS), breast cancer specific-survival (BCSS) and overall survival (OS).

			Zinc quartile	Serum zinc[a]					
				Women (n)	Events (n)	Total person years	Mortality/1000	HR (95% CI)	HR (95% CI)[b]
All		RFS	1	236	52	2616	19.88	1.00	1.00
			2+3+4	703	178	8448	14.83	1.01 (0.74-1.37)	0.95 (0.70-1.31)
		BCSS	1	236	48	2692	17.83	1.00	1.00
			2+3+4	703	128	8630	14.83	0.83 (0.60-1.16)	0.79 (0.56-1.12)
		OS	1	236	89	2692	33.06	1.00	1.00
			2+3+4	703	280	8630	32.44	0.97 (0.77-1.24)	0.88 (0.69-1.13)
Serum phosphorus[c]	Low	RFS	1	72	18	1096	16.42	1.00	1.00
			2+3+4	219	72	3138	22.94	1.43 (0.85-2.40)	1.22 (0.71-2.10)
		BCSS	1	72	11	1111	9.90	1.00	1.00
			2+3+4	219	45	3199	14.07	1.42 (0.74-2.75)	1.09 (0.54-2.19)
		OS	1	72	25	1111	22.50	1.00	1.00
			2+3+4	219	87	3199	27.20	1.22 (0.78-1.90)	0.98 (0.61-1.56)
	High	RFS	1	74	14	1009	13.88	1.00	1.00
			2+3+4	216	69	2955	23.35	1.57 (0.88-2.80)	1.72 (0.93-3.17)
			P_i[e] high versus low phosphorus					0.88	0.95
		BCSS	1	74	19	1033	18.39	1.00	1.00
			2+3+4	216	51	3013	16.93	0.91 (0.54-1.55)	0.89 (0.50-1.59)
			P_i high versus low phosphorus					0.31	0.73
		OS	1	74	32	1033	30.98	1.00	1.00
			2+3+4	216	116	3013	38.50	1.25 (0.85-1.85)	1.24 (0.82-1.89)
			P_i high versus low phosphorus					0.95	0.51
Serum selenium[d]	Low	RFS	1	161	41	1718	23.86	1.00	1.00
			2+3+4	309	84	3584	23.44	0.99 (0.68-1.44)	0.77 (0.51-1.14)
		BCSS	1	161	35	1777	19.70	1.00	1.00
			2+3+4	309	63	3637	17.32	0.88 (0.58-1.33)	0.74 (0.48-1.15)
		OS	1	161	65	1777	36.58	1.00	1.00
			2+3+4	309	137	3637	37.67	1.02 (0.76-1.37)	0.83 (0.61-1.14)
	High	RFS	1	75	11	898	12.25	1.00	1.00
			2+3+4	394	94	4863	19.33	1.50 (0.80-2.80)	1.65 (0.86-3.18)
			P_i high versus low selenium					0.25	0.13
		BCSS	1	75	62	915	67.76	1.00	1.00
			2+3+4	394	329	4992	65.91	0.92 (0.51-1.67)	1.31 (0.67-2.59)
			P_i high versus low selenium					0.91	0.52
		OS	1	75	24	915	26.23	1.00	1.00
			2+3+4	394	143	4992	28.65	1.09 (0.71-1.68)	1.31 (0.81-2.11)
			P_i high versus low selenium					0.81	0.41

[a] Serum zinc quartiles as in Supplementary Table 3.

[b] Adjusted for age at baseline, age at diagnosis, baseline year, tumor size, lymph node status, distant metastasis status and intrinsic subtype.

[c] The cut-off is set at the median. Low and high serum phosphorus is defined as ≤1.18 mmol/L and >1.18 mmol/L, respectively.

[d] The cut-off is set at the median. Low and high serum selenium is defined as ≤90.30 µg/day and >90.30 µg/day, respectively.

[e] P-value for interaction.

Table S5. Zinc intake from foods in relation to recurrence free survival (RFS), breast cancer specific-survival (BCSS) and overall survival (OS).

	Zinc quartile	Zinc intake from foods[a]					
		Women (*n*)	Events (*n*)	Total person years	Mortality/1000	HR (95% CI)	HR (95% CI)[b]
RFS	1	265	73	2989	24.42	1.00	1.00
	2	266	65	3174	20.48	0.82 (0.58-1.14)	0.80 (0.57-1.14)
	3	265	60	3283	18.28	0.70 (0.50-0.98)	0.73 (0.51-1.04)
	4	266	70	3186	21.97	0.89 (0.64-1.24)	0.96 (0.68-1.34)
	P-trend					0.35	0.69
BCSS	1	265	64	3043	21.03	1.00	1.00
	2	266	45	3258	13.81	0.66 (0.45-0.97)	0.69 (0.46-1.05)
	3	265	46	3342	13.76	0.66 (0.45-0.96)	0.75 (0.50-1.12)
	4	266	50	3278	15.25	0.73 (0.50-1.06)	0.78 (0.52-1.16)
	P-trend					0.10	0.26
OS	1	265	116	3043	38.12	1.00	1.00
	2	266	100	3258	30.69	0.80 (0.61-1.05)	0.85 (0.64-1.13)
	3	265	105	3342	31.42	0.81 (0.62-1.05)	0.90 (0.68-1.18)
	4	266	112	3278	34.17	0.88 (0.68-1.15)	1.02 (0.77-1.34)
	P-trend					0.40	0.85

[a] Residuals are presented as the median and interquartile range of total dietary intake of zinc; 1 (8.4 (2.6) mg/day)), 2 (8.8 (2.5)), 3 (10.1 (2.7)) and 4 (12.5 (3.0)).

[b] Adjusted for age at baseline, age at diagnosis, baseline year, tumor size, lymph node status, distant metastasis status and intrinsic subtype.

Table S6. Zinc in diet and serum in relation to recurrence free survival (RFS), breast cancer specific-survival (BCSS) and overall survival (OS), stratified for time between baseline and diagnosis.

		Zinc quartile	Dietary intake of zinc[a]						Serum zinc[a]					
			Women (*n*)	Events (*n*)	Total person years	Mortality/1000	HR (95% CI)	HR (95% CI)[b]	Women (*n*)	Events (*n*)	Total person years	Mortality/1000	HR (95% CI)	HR (95% CI)[b]
< 10.5 years between baseline and diagnosis	RFS	1	127	37	1773	20.87	1.00	1.00	89	24	1433	16.75	1.00	1.00
		2	137	46	2171	21.19	0.98 (0.63–1.52)	0.96 (0.61–1.51)	111	41	1594	25.72	1.56 (0.94–2.58)	1.29 (0.77–2.15)
		3	129	43	1932	22.26	1.01 (0.65–1.57)	1.08 (0.69–1.70)	119	35	1741	20.10	1.14 (0.68–1.93)	1.06 (0.62–1.83)
		4	135	41	2034	20.16	1.06 (0.67–1.65)	1.09 (0.69–1.73)	123	37	1908	19.39	1.11 (0.66–1.86)	1.10 (0.64–1.88)
		P-trend					0.78	0.59					0.82	0.98
	BCSS	1	127	37	1788	20.69	1.00	1.00	89	19	1455	13.06	1.00	1.00
		2	137	30	2200	13.64	0.66 (0.41–1.08)	0.60 (0.36–1.01)	111	31	1619	19.15	1.46 (0.82–2.58)	1.07 (0.60–1.93)
		3	129	25	1985	12.59	0.61 (0.37–1.01)	0.65 (0.38–1.09)	119	28	1778	15.75	1.20 (0.67–2.15)	1.03 (0.56–1.92)
		4	135	29	2077	13.96	0.67 (0.41–1.10)	0.72 (0.44–1.19)	123	23	1953	11.78	0.89 (0.49–2.15)	0.78 (0.41–1.47)
		P-trend					0.11	0.25					0.46	0.40
	OS	1	127	70	1788	39.15	1.00	1.00	89	19	1455	13.06	1.00	1.00
		2	137	62	2200	28.18	**0.71 (0.50–1.00)**	0.75 (0.52–1.07)	111	31	1619	19.15	1.49 (0.98–2.27)	1.27 (0.83–1.95)
		3	129	62	1985	31.23	0.78 (0.55–1.10)	0.81 (0.57–1.15)	119	28	1778	15.75	1.37 (0.90–2.09)	1.17 (0.75–1.81)
		4	135	66	2077	31.78	0.81 (0.58–1.13)	0.86 (0.61–1.21)	123	23	1953	11.78	1.31 (0.87–1.99)	1.12 (0.72–1.73)
		P-trend					0.34	0.49					0.38	0.82
≥ 10.5 years between baseline and diagnosis	RFS	1	139	30	1210	24.79	1.00	1.00	147	28	1183	23.67	1.00	1.00
		2	128	22	1125	19.56	0.76 (0.44–1.31)	0.74 (0.41–1.34)	127	19	1167	16.28	0.64 (0.36–1.15)	0.61 (0.34–1.11)
		3	136	30	1201	2498	0.99 (0.60–1.64)	1.09 (0.64–1.85)	113	22	1086	20.26	0.72 (0.41–1.27)	0.70 (0.40–1.25)
		4	131	19	1186	16.02	0.58 (0.32–1.03)	0.55 (0.30–1.01)	110	24	951	25.24	1.01 (0.59–1.74)	1.00 (0.57–1.75)
		P-trend					0.15	0.14					0.96	1.00
	BCSS	1	139	22	1252	17.57	1.00	1.00	147	29	1237	23.44	1.00	1.00
		2	128	18	1165	15.45	0.88 (0.47–1.64)	1.16 (0.57–2.35)	127	14	1198	11.69	**0.51 (0.27–0.96)**	**0.42 (0.20–0.87)**
		3	136	28	1231	22.75	1.30 (0.74–2.27)	**1.89 (1.00–3.59)**	113	16	1097	14.59	0.65 (0.35–1.19)	0.65 (0.32–1.30)
		4	131	16	1223	13.08	0.76 (0.40–1.44)	0.66 (0.32–1.36)	110	16	985	16.24	0.71 (0.38–1.30)	0.82 (0.42–1.61)
		P-trend					0.75	0.56					0.27	0.56
	OS	1	139	44	1252	35.14	1.00	1.00	147	54	1237	43.65	1.00	1.00
		2	128	41	1165	35.19	0.99 (0.65–1.52)	1.07 (0.67–1.70)	127	35	1198	29.22	0.67 (0.43–1.02)	**0.51 (0.32–0.82)**
		3	136	51	1231	41.43	1.18 (0.79–1.76)	**1.76 (1.14–2.73)**	113	34	1097	30.99	0.70 (0.46–1.08)	0.68 (0.43–1.09)
		4	131	37	1223	30.25	0.86 (0.55–1.33)	0.88 (0.56–1.40)	110	35	985	35.53	0.81 (0.53–1.24)	0.78 (0.49–1.23)
		P-trend					0.72	0.88					0.31	0.37

[a] Serum zinc quartiles and quartiles of dietary intake of zinc as in Table 2.

[b] Adjusted for age at baseline, age at diagnosis, baseline year, tumor size, lymph node status, distant metastasis status and intrinsic subtype.

Table S7. Zinc in diet and serum in relation to 5 and 10-years recurrence free survival (RFS), breast cancer specific-survival (BCSS) and overall survival (OS).

		Zinc quartile	Dietary intake of zinc[a]						Serum zinc[a]					
			Women (*n*)	Events (*n*)	Total person years	Mortality/1000	HR (95% CI)	HR (95% CI)[b]	Women (*n*)	Events (*n*)	Total person years	Mortality/1000	HR (95% CI)	HR (95% CI)[b]
5-years survival	RFS	1	266	19	1213	15.66	1.00	1.00	236	17	1072	15.86	1.00	1.00
		2	265	14	1253	11.17	0.71 (0.36-1.42)	0.68 (0.33-1.40)	238	13	1120	11.61	0.73 (0.36-1.50)	0.67 (0.32-1.43)
		3	265	23	1216	18.91	1.21 (0.66-2.22)	1.38 (0.74-2.57)	232	14	1087	12.88	0.81 (0.40-1.64)	0.83 (0.39-1.75)
		4	266	11	1259	8.74	0.56 (0.27-1.17)	0.57 (0.27-1.23)	233	14	1093	12.81	0.81 (0.40-1.63)	0.70 (0.33-1.47)
		P-trend					0.37	0.53					0.61	0.45
	BCSS	1	266	28	1222	22.91	1.00	1.00	236	28	2692	10.40	1.00	1.00
		2	265	18	1254	14.35	0.66 (0.37-1.18)	0.54 (0.29-1.02)	238	18	2817	6.39	0.62 (0.34-1.12)	0.56 (0.29-1.06)
		3	265	21	1220	17.21	0.97 (0.57-1.64)	1.16 (0.67-2.02)	232	19	2875	6.60	0.68 (0.38-1.21)	0.64 (0.33-1.25)
		4	266	25	1265	19.76	0.62 (0.34-1.12)	0.62 (0.33-1.15)	233	17	2938	5.79	0.60 (0.33-1.09)	0.57 (0.29-1.09)
		P-trend					0.27	0.47					0.11	0.11
	OS	1	266	42	1222	34.37	1.00	1.00	236	37	2692	13.74	1.00	1.00
		2	265	31	1254	24.72	0.78 (0.49-1.24)	0.72 (0.45-1.17)	238	29	2817	10.29	0.75 (0.46-1.23)	0.60 (0.35-1.02)
		3	265	36	1220	29.51	0.93 (0.60-1.44)	1.18 (0.75-1.86)	232	30	2875	10.43	0.81 (0.50-1.30)	0.75 (0.44-1.30)
		4	266	37	1265	29.25	0.80 (0.51-1.26)	0.88 (0.55-1.41)	233	31	2938	10.55	0.82 (0.51-1.32)	0.82 (0.49-1.39)
		P-trend					0.48	0.93					0.48	0.64
10-years survival	RFS	1	266	36	2150	16.74	1.00	1.00	236	32	1882	17.00	1.00	1.00
		2	265	28	2242	12.49	0.74 (0.45-1.22)	0.75 (0.45-1.25)	238	27	1999	13.51	0.79 (0.48-1.32)	0.69 (0.40-1.16)
		3	265	40	2180	18.35	1.10 (0.70-1.72)	1.25 (0.79-1.98)	232	28	1962	14.27	0.84 (0.50-1.39)	0.81 (0.48-1.37)
		4	266	27	2246	12.02	0.72 (0.44-1.18)	0.72 (0.43-1.19)	233	26	1954	13.31	0.78 (0.46-1.31)	0.81 (0.47-1.38)
		P-trend					0.47	0.58					0.40	0.55
	BCSS	1	266	43	2178	19.74	1.00	1.00	236	39	1917	20.34	1.00	1.00
		2	265	31	2264	13.69	0.69 (0.44-1.10)	0.68 (0.42-1.10)	238	32	2021	15.83	0.78 (0.49-1.25)	0.64 (0.38-1.06)
		3	265	46	2199	20.92	1.06 (0.70-1.61)	1.34 (0.87-2.07)	232	32	1969	16.25	0.80 (0.50-1.28)	0.73 (0.43-1.23)
		4	266	31	2283	13.58	0.69 (0.43-1.09)	0.72 (0.45-1.16)	233	28	1985	14.10	0.70 (0.43-1.13)	0.71 (0.42-1.19)
		P-trend					0.37	0.70					0.17	0.25
	OS	1	266	68	2178	31.22	1.00	1.00	236	63	1917	32.86	1.00	1.00
		2	265	61	2264	26.94	0.86 (0.61-1.22)	0.91 (0.64-1.31)	238	55	2021	27.21	0.83 (0.58-1.19)	**0.65 (0.44-0.96)**
		3	265	78	2199	35.47	1.14 (0.82-1.57)	**1.44 (1.03-2.01)**	232	59	1969	29.96	0.91 (0.64-1.30)	0.84 (0.57-1.24)
		4	266	60	2283	26.28	0.84 (0.59-1.19)	0.93 (0.65-1.32)	233	56	1985	28.21	0.86 (0.60-1.23)	0.84 (0.57-1.23)
		P-trend					0.69	0.68					0.52	0.66

[a] Serum zinc quartiles and quartiles of dietary intake of zinc as in Table 2.

[b] Adjusted for age at baseline, age at diagnosis, baseline year, tumor size, lymph node status, distant metastasis status and intrinsic subtype.
